# Special Issue “Physiology and Pathophysiology of the Placenta”

**DOI:** 10.3390/ijms25073594

**Published:** 2024-03-22

**Authors:** Giovanni Tossetta

**Affiliations:** Department of Experimental and Clinical Medicine, Università Politecnica delle Marche, 60126 Ancona, Italy; g.tossetta@univpm.it

The placenta is a transient but essential organ for normal in utero development, playing several essential functions in normal pregnancy [1,2,3,4,5]. The important role of the placenta during pregnancy is highlighted when placental development is impaired, leading to the development of pregnancy complications such as gestational trophoblastic diseases (GTD), gestational diabetes mellitus (GDM), preeclampsia (PE), preterm birth (PTB) and intrauterine growth restriction (IUGR) [6,7,8,9].

In this Special Issue, articles (6) and reviews (5) addressing the major problems in the physiology and pathophysiology of the placenta have been selected for publication.

The study by Peñailillo and colleagues evaluated the role of forkhead box M1 (FOXM1), a transcription factor involved in angiogenesis and cell migration [10,11,12] in trophoblast invasion, identifying that FOXM1 expression is significantly higher in trophoblast cells exposed to hypoxia (3% O_2_), while its expression significantly decreases under more tight hypoxic conditions (1% O_2_). FOXM1 overexpression in HTR-8/SVneo cells increases their migration and tubule formation ability. Moreover, trophospheres obtained from a 3D culture show higher FOXM1 expression compared to pre-invasion trophospheres. Furthermore, FOXM1-depletion in HTR-8/SVneo cells leads to the downregulation of Polo-Like Kinase 4 (PLK4), Vascular Endothelial Growth Factor (VEGF), and Matrix Metallopeptidase 2 (MMP-2) mRNA expression demonstrating how FOXM1 participates in embryo implantation by favoring early trophoblast invasion.

Placenta accreta spectrum (PAS) is an important pregnancy complication caused by an excessive invasion of cytotrophoblast cells in the endometrial–myometrial interface [13,14]. Timofeeva and colleagues reported how the evaluation of the serum levels of miR-26a-5p, miR-17-5p, and miR-101-3p in the first trimester demonstrated 100% sensitivity in detecting PAS. Thus, the detection of these miRNAs can serve as an auxiliary method for the first-trimester screening of pregnant women. The importance of evaluating miRNAs during pregnancy has also been highlighted in the review article published by Giannubilo and colleagues.

PE occurs in 5–7% of pregnancies and is generally diagnosed in the second half of pregnancy when its clinical manifestations (proteinuria and hypertension) occur [15,16,17]. Shallow trophoblast invasion in the endometrium/myometrium characterizing PE is also the cause of the occurrence of a hypoxic environment, which leads to increased oxidative stress [18,19]. Oxidative stress is involved in several complications and diseases, including cancer, neurodegenerative diseases and endothelial dysfunction [20,21,22,23,24,25,26].

Antihypertensive therapy is essential for the management of patients with PE, and methyldopa (Dopegyt^®^) and nifedipine (Cordaflex^®^) are the common drugs used to stabilize blood pressure [27,28]. The study by Ziganshina and colleagues analyzed the effect of antihypertensive therapy on the expression of fucosylated glycans in the fetal capillaries of placental terminal villi in patients with early-onset PE (EOPE; onset < 34 weeks of gestation) and late-onset PE (LOPE; onset ≥ 34 weeks of gestation). The authors found that the expression patterns of fucosylated glycans in endothelial glycocalyx (eGC) in the terminal villi of EOPE and LOPE are characterized by the predominant expression of structures with a type-2 core due to one or both being antihypertensive drugs. These changes in eGC fucoglycans were in accordance with maternal hemodynamics, fetoplacental hemodynamics, and humoral factors associated with eGC damage, demonstrating the effects of antihypertensive therapy on placental eGC in women with PE.

The study by Filippi and colleagues evaluated umbilical venous and arterial oxygen levels, fetal oxygen extraction, oxygen content, CO_2_, and lactate in healthy newborns with gestational age < 37 weeks and found a progressive decrease in oxygen levels associated with a concomitant increase in CO_2_ levels and reduction in pH starting from the 23rd week to the 33rd–34th week of gestation. From the 33rd–34th week onwards, fetal oxygenation increased, demonstrating that oxygenation during intrauterine life continues to vary even after placenta development.

Placental protein 13 (PP13) is a key protein involved in vascular remodeling and immune tolerance [29,30,31]. Kazatsker and colleagues evaluated soluble and placental-associated extracellular vesicles (PEV-associated PP13) in the maternal uterine vein in normal, PE and preterm conditions. The authors found that soluble PP13 was not significantly altered across PE, preterm and term delivery, but after depleting the PEV of their proteome, the total PP13 (soluble and PEV-associated PP13) was increased in preterm PE (but not in cases of preterm or term delivery). Corticosteroid treatment caused a depletion of PP13 from the PEV, especially in preterm PE patients.

The functions of annexin A1 (ANXA1) have a key role in protecting cells against DNA damage [32,33]. The study by Moreli and colleagues found that, in ANXA1 knockout mice (AnxA1−/−), the labyrinth zone of the placenta was reduced, and there was increased DNA damage, causing apoptosis in the labyrinthine and junctional layers. In pregnant women with gestational diabetes mellitus (GDM), placenta AnxA1 expression was reduced, and there was increased DNA damage and apoptosis, suggesting the possible involvement of ANXA1 in oxidative DNA damage response under hyperglycemia.

In addition to these insightful research articles, two reviews in this Special Issue highlight the role of uterine receptivity and point mutations in recurrent miscarriage (reviewed by Günther and colleagues) and pregnancy loss (reviewed by Maksiutenko and colleagues), respectively. Furthermore, Berna-Erro and colleagues review the main platelet modifications during hypoxia in order to highlight new platelet markers of hypoxic conditions during labor.

Finally, the review by Benagiano and colleagues highlights the nature of maternal–embryonic communication and the major mechanisms active during the pre-implantation period in order to better understand how the placenta forms during pregnancy.

The eleven articles published in this Special Issue prove the growing interest in finding new molecular targets and mechanisms involved in the regulation of placental development and new markers to predict pregnancy complications. We hope to provide our readers with a new representative and useful snapshots of the current problems in placental development in order to inspire new studies in this field. I personally acknowledge all the contributors of this Special Issue, the Editorial Board, and the assistant editors of the *International Journal of Molecular Sciences* for their support.
